# Saudi Healthcare Students’ General Practices Applied to Protect Against COVID-19 and Their Beliefs Regarding the Use of Herbal Supplements as a Protection Method

**DOI:** 10.7759/cureus.53959

**Published:** 2024-02-10

**Authors:** Raid M Al Zhranei, Fenoon Aljohani, Samaher Almutairi, Sawsan Abdulhafiz, Wafaa Aljohani

**Affiliations:** 1 Department of Respiratory Therapy, College of Applied Medical Sciences, King Saud bin Abdulaziz University for Health Sciences, Jeddah, SAU; 2 Research Office, King Abdullah International Medical Research Center (KAIMRC), Jeddah, SAU; 3 Department of Respiratory Therapy, College of Applied Medical Sciences, King Saud Bin Abdulaziz University for Health Sciences, Jeddah, SAU; 4 Department of Medical-Surgical Nursing, Faculty of Nursing, King Abdulaziz University, Jeddah, SAU; 5 Department of Nursing, Batterjee Medical College, Jeddah, SAU

**Keywords:** herbal research, corona virus disease 2019, protection, herbal supplements, covid-19

## Abstract

Background: At the beginning of the COVID-19 pandemic, the absence of treatment increased the Saudi population's inquietude regarding the virus. Therefore, people were seeking alternative methods to protect themselves from disease's fast transmission, such as hand hygiene, social isolation, and the use of natural and dietary products.

Aim: The main objective of this study is to assess healthcare students’ general practices applied to protect against COVID-19 and their beliefs regarding the use of herbal supplements as a protection method.

Methodology: All undergraduate healthcare students were eligible to participate, except pre-professional students. The estimated sample size was 371, which was calculated using Raosoft® software (Raosoft Inc., Seattle, WA). A cross-sectional online survey was distributed among the targeted population. The data were entered in Microsoft Excel (Microsoft Corporation, Redmond, WA) and transferred to be analyzed by JMP software (SAS Institute Inc., Cary, NC).

Results: A total of 441 healthcare students, who met the inclusion criteria, participated in this study. About 81.41% of the participants reported that they were completely committed to Saudi guidelines regarding preventive measures against COVID-19 transmission. Most of the participants were not using herbal supplements, while 17.1% of them used herbal products or dietary supplements during the COVID-19 pandemic. The participants who used herbal and dietary products commonly consumed ginger and vitamins C and D. In addition, a chi-square showed significant differences in gender and specialties regarding the usage of herbal supplements as a protective method against COVID-19 (P<0.05).

Conclusion: The findings of our study exhibit the general practice of herbal products during the COVID-19 pandemic among Saudi healthcare students was low in comparison with their beliefs. In addition, the using of herbal supplements should be evidence-based to guarantee safe consumption.

## Introduction

At the end of 2019, several pneumonia cases were reported in Wuhan’s local hospital, in China, with unknown etiology. The scientists conducted many investigations to understand the genetic sequence of this unknown cause. After investigations, the scientists discovered that this unknown pathogen belonged to the coronavirus’s family, which was named as a novel coronavirus (2019-nCoV). Consequently, in March 2020, the virus spread rapidly globally and was announced as an outbreak of pandemic by the World Health Organization (WHO). The first case of COVID-19 in Saudi Arabia was announced by the Ministry of Health (MOH) in early March 2020 [1-6].

Worldwide, to protect the communities from the spread of the virus, many countries have developed guidelines to restrict the transition of COVID-19, thus reduce the pressure on the healthcare system. In Saudi Arabia, the Ministry of Health (MOH) recommended applying strict hygienic standards, limiting social gatherings, and working on and strengthening the immune system [7-10].

The Saudi population’s concerns and fears of being infected with COVID-19 have increased due to the absence of the virus treatment, so finding an adjuvant treatment was required. As a consequence, several people used herbal supplements as alternative methods, whose effects have been proven according to their phytotherapy. The consumed herbs are known for their effectiveness in boosting the immune system and treating respiratory symptoms, such as coughing, chest pain, and shortness of breath [1,11,12].

Many studies mentioned the possibility of using herbal supplements due to their unique antioxidants and anti-inflammatory components that will activate the immune system and lower the risk of inflammatory lung diseases, such as frankincense, Curcuma, and garlic [8,12-14].

Therefore, several countries utilized their traditional herbal supplements in COVID-19 cases [14]. Although the usage of herbal supplements is widely spread, a systematic review highlighted the interaction between the herbal supplements and the drugs, either synergism or antagonism, in addition to the severity of this interaction, which could be life-threatening. Hence, the consumers of herbal products should be aware of herbs-drugs interactions [15].

In addition to the herbal products, dietary supplements were used to strengthen the immune system. There is an inverse correlation between immunodeficiency and vitamins; thus, people with vitamin deficiency are at high risk of developing diseases. Dietary supplements play an integral role in the inhibition of inflammatory mediators such as cytokines, chemokines, and leukotrienes. Vitamin D and C, as examples of dietary supplements, have anti-inflammatory and antioxidant effects. 1,25-dihydroxyvitamin D metabolism stimulates the type II alveolar cells to produce surfactant, which is an important component in the lungs [16,17].

During of COVID-19 pandemic, several studies discussed the usage of herbal supplements in combination with conventional treatments to decrease COVID-19 complications [8,13].

Moreover, a study was conducted to investigate the prevalence of herb consumption among the Saudi community during the pandemic, since individuals who utilized herbs to protect themselves from the virus were noticed [1].

This study aims to identify healthcare students’ general practices applied to protect against COVID-19 and their beliefs regarding the use of herbal supplements as a protection method. In addition, to measure other sociodemographic variables that may influence in using of herbal products.

## Materials and methods

A cross-sectional descriptive study was conducted between February and December 2021 through an online questionnaire. The inclusion criteria for the study include all healthcare students of King Saud bin Abdulaziz University for Health Sciences (KSAU-HS) in its three regions, Riyadh, Jeddah, and Al-Ahsa in Saudi Arabia. Meanwhile, pre-healthcare students who were first-year students were excluded from the study.

In addition, all healthcare students at King Saud bin Abdulaziz University for Health Sciences are roughly about 10,395 [18]. Consequently, the required sample size according to Raosoft® software by the website http://www.raosoft.com/samplesize.html (Raosoft Inc., Seattle, WA) was estimated at the 95% confidence level with a margin of error of ±5%. Then the required sample size was determined to be 371.

The proposal of this study was revised and approved by the ethics committee of King Abdullah International Medical Research Center (KAIMRC) with IRB number SP21J/102/03, then the questionnaire (see Appendices) was administered non-probably using the convenience sampling technique to the targeted population through social media applications (Twitter and WhatsApp). A consent form was obtained before answering the questionnaire; thus, all participants were notified that their participation was voluntary and all responses would be kept anonymous.

The permission was taken to adapt a previous study published survey from Alyami et al [1]. Their instrument was used in Saudi Arabia between May and June 2020 to investigate the Saudi population's knowledge and beliefs regarding the consumption of herbal supplements to prevent COVID-19 infection. Three qualified researchers reviewed the instrument's validity and reliability. The tool included 22 questions, which were divided into four sections. The first section assessed the demographic characteristics of the participants, such as gender, age, specialty, and academic year. The second section included questions to assess the participants' knowledge about preventive measures against COVID-19. The third section assessed the practices of using herbal and dietary supplements among healthcare students as a protective method. The last section evaluated the participants' beliefs regarding herbal products and dietary supplements to protect against COVID-19 [1]. In addition, some modifications were made in the demographic section to suit the study's target population.

Statistical analysis

After completing data collection, the data were entered using Microsoft Excel (Microsoft Corporation, Redmond, WA) and processed by using JMP software trial version 15.2.1 (482026) (SAS Institute Inc., Cary, NC). All of the data were quantitative and reported as frequency and percentages. Moreover, a chi-square test as an inferential statistic was used to analyze the data and examine the differences between group variables. A confidence interval of 95% (p<0.05) was applied to represent the statistical significance of the results, and the level of significance was predetermined as 5%.

## Results

Demographic characteristics data

A total of 441 healthcare students who met the inclusion criteria participated in this study. Around 278 (63.04%) were females, 244 (55.33%) studying on the Jeddah campus, and 374 (84.81%) were aged between 20 and 23 years. Around 156 (35.37%) of the students were in their fourth year, and 125 (28.34%) of them were in the respiratory therapy specialty. In addition, (75.06%) of the respondents were not infected by COVID-19 (Table 1).

**Table 1:** Demographic characteristics of the study participants (n=441).

Variables	n (%)
Gender	
Male	163 (36.96)
Female	278 (63.04)
Age	
19 or less	51 (11.56)
20 to 23	374 (84.81)
24 to 27	16 (3.63)
Campus	
Riyadh	135 (30.61)
Jeddah	244 (55.33)
Al-Ahsa	62 (14.06)
Academic year	
Second-year	61 (13.83)
Third-year	123 (27.89)
Fourth-year	156 (35.37)
Fifth-year and above	101 (22.90)
Specialty	
Medicine	89 (20.18)
Respiratory therapy	125 (28.34)
Emergency medical services	22 (4.99)
Occupational therapy	35 (7.94)
Radiological sciences	23 (5.22)
Pharmacology	36 (8.16)
Nursing	54 (12.24)
Other	57 (12.93)
Being infected with COVID-19	
Yes	94 (21.32)
No	331 (75.06)
Maybe	13 (2.95)
Don’t know	3 (0.68)

COVID-19 preventive measures

Regarding COVID-19 preventive measures, 394 (89.34%) of participants agreed that washing hands with soap and water reduces the spread of COVID-19. However, only 153 (34.69%) knew the correct steps of washing hands, which are five steps. Most of the participants 359 (81.41%) reported that their commitment to stay-at-home and avoid gatherings contributed to reducing COVID-19 transmission, and 294 (66.67%) of them knew that the appropriate social distance needed to avoid virus transmission, which is two meters. Moreover, 205 (46.49%) reported that they are completely committed to the Ministry of Health's instructions regarding the COVID-19 pandemic. For more information, see Table 2. 

**Table 2:** Knowledge about COVID-19 preventive measures.

Knowledge	n (%)
Do you think washing hands with soap and water reduces the spread of COVID-19?	
Yes	394 (89.34)
No	14 (3.17)
Maybe	33 (7.48)
How long should you wash your hands with soap and water (in seconds)?	
5	27 (6.12)
10	96 (21.77)
20	180 (40.82)
40	138 (31.29)
How many steps for washing hands with soap and water?	
4	53 (12.02)
5	153 (34.69)
6	141 (31.97)
Don’t know	94 (21.32)
Do you think the commitment to stay-at-home and avoid gathering contributes to reducing COVID-19 transmission?	
Yes	359 (81.41)
No	19 (4.31)
Maybe	62 (14.06)
Don’t know	1 (0.23)
How far should you be away from others to protect yourself from COVID-19 transmission (in meters)?	
0.5	16 (3.63)
1	120 (27.21)
2	294 (66.67)
5	11 (2.49)
Do you consider yourself complied with the instructions of the Saudi Ministry of Health?	
Totally complied	205 (46.49)
Yes, except in cases of extreme necessity	146 (33.11)
To some extent	81 (18.37)
No	9 (2.04)

The usage and beliefs of herbal products and dietary supplements to protect against COVID-19

Around 75 (17.00%) of the participants reported that they have used herbal products or dietary supplements during the COVID-19 pandemic. Nearly half, 59 (49.58%) of them were motivated to use these herbal products by friends and relatives. The most common herbal products used were ginger 70 (15.87%), black seeds 65 (14.74%), and garlic 45 (10.20%). Regarding the common dietary supplements used by all the participants, 231 (52.38%) of them used vitamin C (Table 3).

**Table 3:** The usage of herbal products and dietary supplements to protect against COVID-19.

Usage	n (%)	
Did you use herbs to protect yourself from COVID-19?		
Yes	75 (17.00)	
No	322 (73.02)	
I used to, but I stopped	44 (9.98)	
In case of Yes and used to, who recommended you to use herbal products? (n=119)		
Internet websites/social media	28 (23.53)	
Friends/relatives	59 (49.58)	
Dietician, physician, pharmacist, nurse, health practitioner	22 (18.49)	
Others	10 (8.40)	
Types of herbs that were used, multiple answers (n=441)	Yes	No
Ginger	70 (15.87)	371 (84.13)
Garlic	45 (10.20)	396 (89.80)
Black seeds	65 (14.74)	376 (85.26)
Turmeric	24 (5.44)	417 (94.56)
Green tea	39 (8.84)	402 (91.16)
Cinnamon	25 (5.67)	416 (94.33)
Anise	24 (5.44)	417 (94.56)
Chamomile	20 (4.54)	421 (95.46)
Others (more than six types)	77 (17.46)	364 (82.54)
Using dietary supplements, multiple answers (n=441)	Yes	No
Vitamin C	231 (52.38)	210 (47.62)
Vitamin D	101 (22.90)	340 (77.10)
Omega 3	77 (17.46)	364 (82.54)
Others (more than three types)	54 (12.24)	387 (87.76)

Around 164 (37.19%) of the participants’ beliefs in using herbal products and dietary supplements to boost immunity and reduce the chance of COVID-19. The findings are summarized in Table 4.

**Table 4:** Beliefs about the use of herbal products and dietary supplements to protect against COVID-19 (n=441).

Beliefs	n (%)			
Question	Yes	No	Maybe	Don’t know
Do you think ginger tea helps to increase immunity and reduce the chance of COVID-19?	125 (28.34)	67 (15.19)	191 (43.31)	58 (13.15)
Do you think eating garlic helps to boost immunity and reduce the risk of being infected?	155 (35.15)	69 (15.65)	173 (39.23)	44 (9.98)
Do you think eating fish oil known as omega-3 helps to boost immunity and protect from COVID-19?	106 (24.04)	73 (16.55)	157 (35.60)	105 (23.81)
Do you think the consumption of vitamin C found in citrus has a role in treating or reducing the chance of developing COVID-19?	204 (46.26)	51 (11.56)	142(32.20)	44 (9.98)
In your opinion, does the consumption of nutritional and herbal supplements prevent the spread of COVID-19 more than social distancing?	50 (11.34)	269 (61.00)	81 (18.37)	41 (9.30)
In your opinion, can vitamin and herbal supplements treat or reduce the incidence of COVID-19?	164 (37.19)	96 (21.77)	146 (33.11)	35 (7.94)

Association between questions and demographic data

Based on the chi-square test, participants significantly differed in gender and academic year regarding the use of herbal supplements as a protective method against COVID-19 (P<0.05). Females were the most users of herbal supplements among participants (P=0.013). In addition, as the academic year increases, the usage of herbal products as a protective method decreases (P=0.0001) (Table 5).

**Table 5:** Association between gender, campuses, and academic year with the use of herbal products (n=441). Significant value <0.05.

Variable	Using herbal products as a protective method against COVID-19					
Gender	Yes, n (%)	No, n (%)	I used to, but I stopped, n (%)	P-value	X^2^	DF
Male	25 (5.67)	130 (29.48)	8 (1.81)	0.0130	8.69	2
Female	50 (11.34)	192 (43.54)	36 (8.16)			
Campuses						
Riyadh	23 (5.22)	99 (22.45)	13 (2.95)	0.2102	5.85	4
Jeddah	40 (9.07)	184 (41.72)	20 (4.54)			
Al-Ahsa	12 (2.72)	39 (8.84)	11 (2.49)			
Academic year						
Second year	7 (1.59)	36 (8.16)	18 (4.08)	0.0001	33.46	6
Third year	27 (6.12)	90 (20.41)	6 (1.36)			
Fourth year	25 (5.67)	117 (26.53)	14 (3.17)			
Fifth year and above	16 (3.63)	79 (17.91)	6 (1.36)			

## Discussion

Our study identified the general practices of healthcare students applied to protect against COVID-19 and their beliefs regarding the usage of herbal products as a protection method. The findings of our study revealed the general practices of healthcare students toward herbal and dietary supplements to protect against COVID-19. The majority of the participants reported that they did not use any herbals or dietary supplements during the pandemic. Regarding the students' beliefs about using herbal products and dietary supplements, the participants moderately believed in the effect of common herbals and dietary supplements in boosting immunity and enhancing general respiratory symptoms. In addition, as the participants are healthcare students, they were well-educated about COVID-19 preventive measures.

To control the significant spread of COVID-19, several countries have developed guidelines for citizens to control and reduce COVID-19 transmission. Quarantine measures were implemented, including the closure of public facilities such as schools, universities, workplaces, and transport stations [5,9,10]. In our study, 46.5% of the participants reported that they were completely committed to Saudi Ministry of Health guidelines to reduce COVID-19 transmission.

Before the vaccination was synthesized, the pandemic paralyzed the healthcare system, which increased concerns about finding an alternative solution to strengthen the immune system, such as herbal products whose effectiveness had been approved according to several studies [11,12,19]. Our study showed that the percentage of healthcare students (26.99%) who reported that they used herbal products and dietary supplements as a protective method against COVID-19 was nearly consistent with the findings of the previous study [1]. On the other hand, the majority of the participants (73.02%) did not use any kind of herbal products or food supplements. However, the healthcare students moderately believed in the ability of herbal products and dietary supplements to promote immunity.

Earlier studies were carried out on the effectiveness of natural products in inflammation lung diseases. Thus, several studies investigated herbal supplements against COVID-19 [11,13,17]. According to that, the participants who used herbal products were consuming natural herbs that were known for their positive influence on the immune system.

Ginger and garlic were the most commonly consumed herbal products, and the belief about their influence in enhancing the immunity against COVID-19 was moderate.

During the pandemic, using of dietary supplements, such as vitamins C and D, was popular among society regarding their defensive role, whose anti-inflammatory and antioxidant effects have been proven by plenty of scientific research [12,16,17]. Since the healthcare students' have pharmacological background regarding dietary supplements, our study findings represent the high usage and belief in vitamin C.

In Arab cultures, utilizing medical plants is popular due to it was the main source of treatment before using biomedicine. In Saudi Arabia, the consumption of herbs relies on cultural and religious factors. Moreover, plenty of studies showed gender differences in using of herbal products. The implementation of natural supplements was most important in the female community, since females encourage and support the usage of natural herbs in different health conditions such as pregnancy and menstrual cycles [20,21]. In this study, the findings showed that female consumption of herbal products as a protective method against COVID-19 was higher than males, which is similar to the previous studies.

The strengths of our study include that it is the first study to measure healthcare students’ general practices and beliefs regarding the use of herbal supplements as a protective method against COVID-19. However, one of the study limitations is that the study was conducted through an online survey, which resulted in the absence of some desirable population. In addition, the use of convenience sampling and the participants being limited to King Saud bin Abdulaziz University for Health Sciences (KSAU-HS) students may limit the generalizability of the findings to the broader population.

## Conclusions

In conclusion, high utilization of natural and dietary supplements during the COVID-19 pandemic was noticed among individuals. Therefore, our study investigated the general practice of herbal products during the COVID-19 pandemic among Saudi healthcare students. However, the findings of our study showed that the majority of the healthcare students reported that they did not use any herbals or dietary supplements during the pandemic. Regarding the healthcare students' beliefs about using herbal products and dietary supplements, they moderately believed in the effect of common herbals and dietary supplements in boosting immunity and enhancing general respiratory symptoms. In addition, the study found that the healthcare students were well-educated about COVID-19 preventive measures. We recommend the spread of knowledge about the positive effect of herbal products in boosting the immune system among healthcare students.

## Appendices

Research questionnaire

Section 1: Demographic Data.

Q1 - Gender:

·         Male

·         Female

Q2 - Age (in years):

·         19 or less

·         20 to 23

·         24 to 27

·         Greater than or equal to 28

Q3 - Campuses:

·         Riyadh

·         Jeddah

·         Al-Ahsa

Q4 - Academic year:

·         1st Year

·         2nd Year

·         3rd Year

·         4th Year

·         5th Year and above

Q5 - Speciality:

·         Medicine

·         Respiratory therapy

·         Emergency medical services

·         Occupational therapy

·         Radiological sciences

·         Pharmacology

·         Nursing

·         Other

Q6 - Have you been infected by COVID-19?

·         Yes

·         No

·         Don’t know

Section 2: Knowledge about COVID-19 preventive measures.

Q7 - Do you think washing hands with soap and water reduces the spread of COVID-19?

·         Yes

·         No

·         Maybe

Q8 - How long should you wash your hands with soap and water (in seconds)?

·         5

·         10

·         20

·         40

Q9 - How many steps for washing hands with soap and water?

·         4

·         5

·         6

·         Don’t know

Q10 - Do you think your commitment to stay-at-home and avoid gatherings contributes in reducing COVID-19 transmission?

·         Yes

·         No

·         Maybe

·         Don’t know

Q11- How far should you be away from others to protect yourself from COVID-19 transmission (meter)?

·         0.5

·         1

·         2

·         5

Q12 - Do you consider yourself complied with the instructions of The Saudi Ministry of Health?

·         Totally complied

·         Yes except in cases of extreme necessity

·         To some extent

·         No

Section 3: The usage of herbal products and dietary supplements to protect against COVID-19.

Q13- Did you use herbs to protect yourself from COVID-19?

·         Yes

·         No

·         I used to, but I stopped

Q14 - If your answer is "Yes," who recommended you to use herbal products?

·         Internet Websites/ Social Media

·         Friends/ relatives

·         Dietician, physician, pharmacist, nurse, health practitioner

·         Others

Q15 - Types of herbs that were used: (You can choose more than one)

·         I didn’t use

·         Ginger

·         Garlic

·         Black seed

·         Turmeric

·         Green tea

·         Cinnamon

·         Anise

·         Oregano

·         Chamomile

·         Sesame seeds

·         *Artemisia absinthium* (Wormwood)

·         Herbal mixtures

·         Licorice

·         Ratany

·         Others

Q16 - What type of dietary supplements did you use before/during the pandemic? (You can choose more than one answer)

·         I didn't use

·         Vitamin C

·         Vitamin D

·         Selenium

·         Royal Jelly

·         Omega 3

·         Other

Section 4: Beliefs about the use of herbal products and dietary supplements to protect against COVID-19.

Q17 - Do you think ginger tea helps to increase immunity and reduce the chance of COVID-19?

·         Yes

·         No

·         Maybe

·         Don’t know

Q18 - Do you think eating garlic helps to boost immunity and reduce the risk of being infected?

·         Yes

·         No

·         Maybe

·         Don’t know

Q19- Do you think that eating fish oil known as omega-3 helps to boost the immunity and protect from COVID-19?

·         Yes

·         No

·         Maybe

·         Don’t know

Q20 - Do you think the consumption of vitamin C found in citrus has a role in treating or reducing the chances of developing COVID-19?

·         Yes

·         No

·         Maybe

·         Don’t know

Q21 - In your opinion, does the consumption of nutritional and herbal supplements prevent the spread of COVID-19 more than social distancing?

·         Yes

·         No

·         Maybe

·         Don’t know

Q22 - In your opinion, can vitamin and herbal supplements treat/reduce the incidence of COVID-19?

·         Yes

·         No

·         Maybe

·         Don’t know
